# Case Report: Could topical epidermal growth factor be considered a new therapy for skin injuries in premature infants?

**DOI:** 10.3389/fped.2024.1424191

**Published:** 2024-06-28

**Authors:** F. Cossovel, F. Marrazzo, S. Nider, A. Traunero, G. Dussi, L. Travan, M. Cirino, D. Zanon

**Affiliations:** ^1^ Department of Neonatology, Institute for Maternal and Child Health, IRCCS “Burlo Garofolo”, Trieste, Italy; ^2^ Department of Medical, Surgical and Health Sciences, University of Trieste, Trieste, Italy; ^3^ Department of Pharmacy, Institute for Maternal and Child Health, IRCCS “Burlo Garofolo”, Trieste, Italy

**Keywords:** preterm, decubit lesion, skin injuries, epithelial growth factor, newborn

## Abstract

In this case report, we present the experience of a premature neonate born at 28 weeks of gestation who, following prolonged respiratory support, developed a pressure injury on the columella despite the implementation of all appropriate preventive techniques. This injury did not improve with standard therapies; therefore, it was necessary to apply a topical galenic therapy containing epidermal growth factor, resulting in complete healing of the lesion.

## Introduction

Nasal injury remains a prevalent issue in very preterm infants undergoing non-invasive respiratory support (1). Before 30 weeks of gestation, the epidermal skin layer and stratum corneum exhibit fragility, elevating the risk of pressure-related skin breakdown in preterm infants. There is limited data in the literature: some studies report an incidence of 20%, which is likely underestimated if we consider the very low birth weight population (2). The most common skin injuries in this population involve the buttocks and facial region, particularly 50% occur at the nasal level (3). These areas are most prone to maceration from diaper use or pressure due to respiratory support (4). Nasal injury typically begins within the first few days of initiating respiratory support (on average 2–3 days), with severe forms appearing around 9–10 days (5). The primary cause of nasal damage associated with CPAP arises from the pressure applied to the columella due to tightly fitted binasal prongs. In the case of nasal lesions from respiratory support, alleviating the pressure is crucial for both healing and prevention. Ensuring the proper sizing of binasal prongs is imperative; prongs that are too small may shift excessively and harm the internal nares, whereas overly large prongs could lead to nasal flaring. The device is frequently changed to redistribute pressure areas or nasal barrier dressings are used. Despite the provision of expert nursing care, encompassing skin monitoring and accurate positioning of the binasal prongs, preventing nasal damage can pose significant challenges, especially in extremely premature or very low birth weight infants. Indeed, in this population, these techniques are not always sufficient, and sometimes skin injuries still occur. When this happens, we face a double problem: the patient's discomfort worsens with prolonged respiratory support, and there is an increased risk of infection associated with the persistence of the skin lesion. While nasal barrier dressings appear effective in reducing CPAP-induced nasal injury, they may not always suffice. Topical application of growth factors are promising in treating various kinds of skin wounds but there are no studies or case report that evaluate its use on newborn or premature.

## Case description

A female infant born at 28 weeks of gestation underwent an emergency cesarean section due to maternal pre-eclampsia and severe fetal growth restriction characterized by reverse flow in the umbilical artery. Continuous positive airway pressure (CPAP) support was initiated immediately after birth and maintained for 41 days, alternating nasal prongs or mask and applying skin barriers to reduce the risk of nasal injury. Oxygen was administered only at birth, a maximum requirement of 30%. Parenteral nutrition was started on the first day of life and maintained for 32 days of life due to poor enteral tolerance. From the second day of life, the baby received breast milk, and total enteral feeding was achieved on the 33th day of life. The newborn never required antibiotic therapy, and nutritional exams, including renal function, liver function, electrolytes, and calcium-phosphorus metabolism, were always normal, except for the finding of cholestatic hepatitis on the 25th day of life, likely secondary to prolonged parenteral nutrition. This resolved with ursodeoxycholic acid therapy.

**Figure 1:**
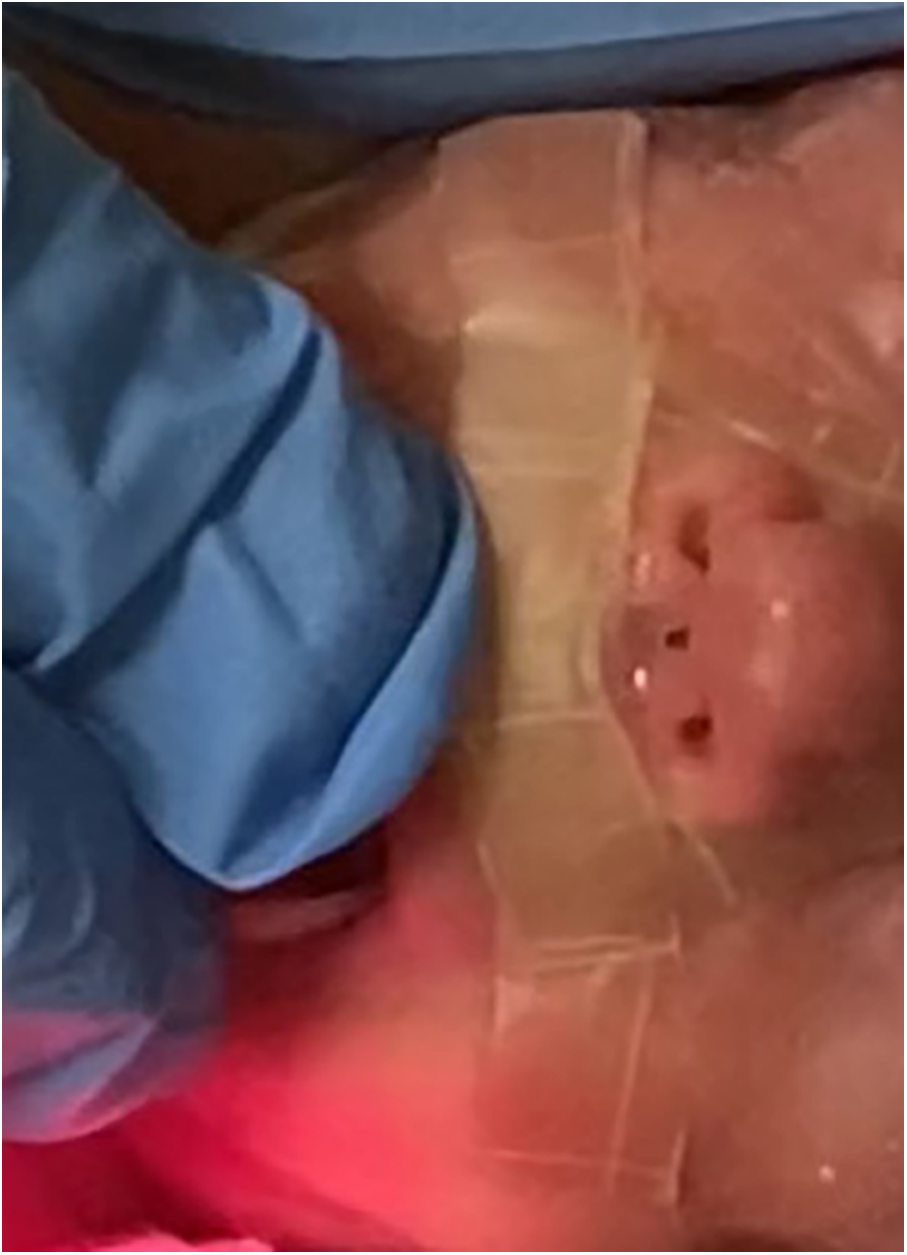
Lesion on the columella at day 28.

**Figure 2:**
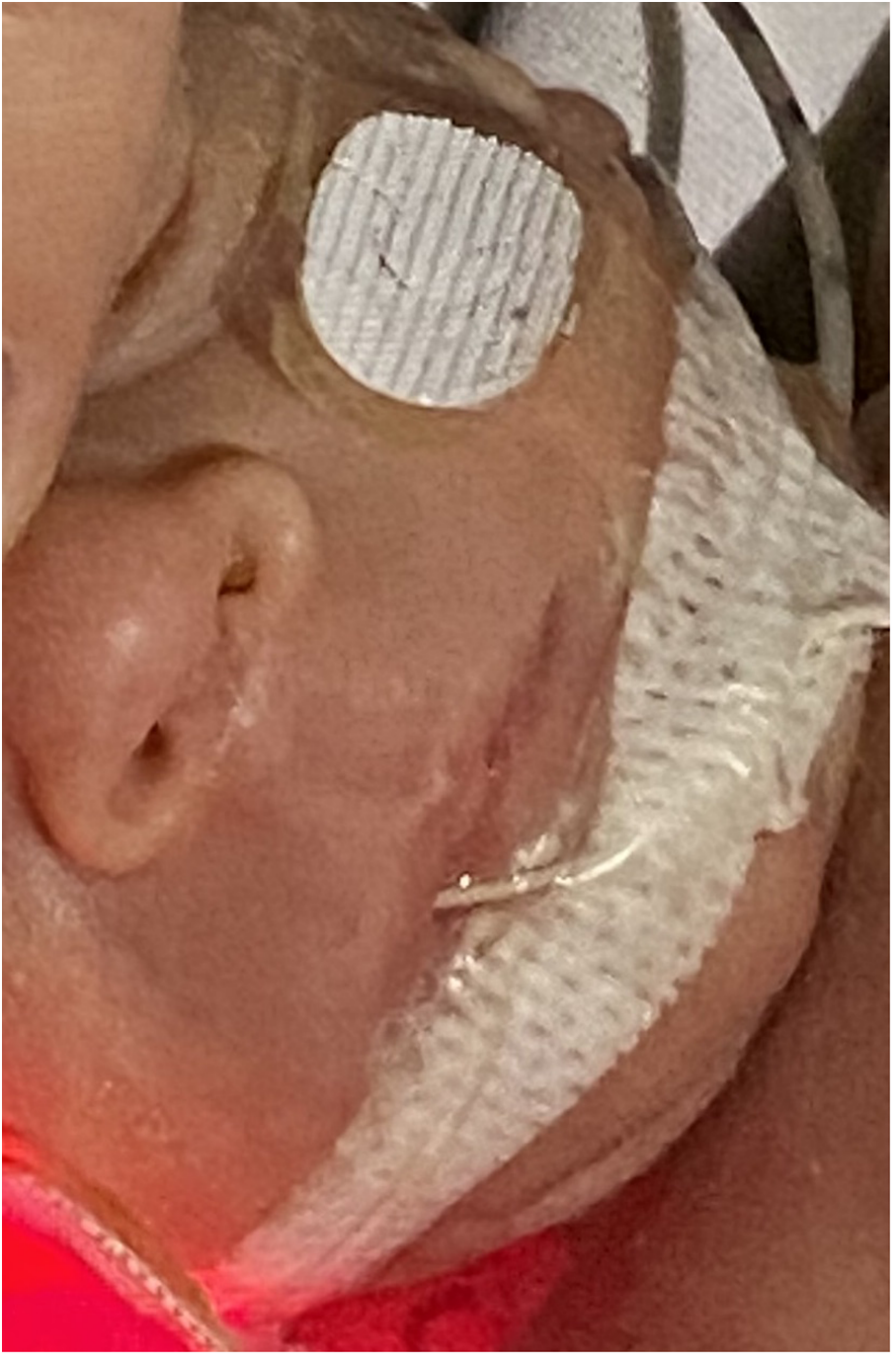
The lesion completely healed after 10 days of topical epidermal growth factor treatment.

On day 14, a lesion on the columella was identified, initially presenting with hyperemia classifiable as stage I according to Fischer et al (5). Topical therapies applied once a day, including hyaluronic acid, hydrogel, collagenase, and clobetasol, were administered without benefit. By day 28, an initial loss of tissue was observed (Figure 1) and a skin lesion stage III according to Fischer et al. (5) was classified. A topical therapy using a gel containing Epidermal Growth Factor (EGF) was administered thrice daily. The gel was applied to the lesion site, and throughout the treatment period, the newborn used appropriate respiratory devices to avoid stressing the injured area. After ten days the lesion exhibited complete healing without any aesthetically displeasing signs (Figure 2).

## Discussion

This is the first case report documenting the use of epidermal growth factor on a pressure injury in a premature neonate. Skin injuries remain a prevalent issue in very preterm infants due to the fragility of the epidermal skin layer and stratum corneum. In such situations, having therapies that allow for effective and rapid healing becomes crucial. A topical galenic therapy-based compound with EGF could be utilized. In this case, the galenic formulation has been obtained using a biological origin EGF. It consists of a 100% characterized 53-amino acid protein obtained from recombinant DNA, identical to human EGF, and stabilized in a suitable non-aqueous vehicle. EGF is a mitogenic protein that stimulates, through interaction with its receptor EGF-R and tyrosine kinase activation, cell growth, proliferation and differentiation, which are essential for tissue repair. EGF plays a significant role in wound healing by promoting cell renewal, and maintaining healthy skin. Additionally, EGF influences various biological processes such as keratinocyte proliferation, fibroblast activation, and angiogenesis (6–8).

The topical use of EGF has been ongoing since 1989 to improve the healing process of various peripheral tissue lesions (9), as well as its intravenous, oral and rectal administration for gastrointestinal damage (10–12). The therapeutic efficacy and tolerability of EGF appear to be demonstrated as the lack of long-term adverse effects was highlighted in the same studies with 6, 12 and 24 months of patient follow-up (13). Convincing experimental data indicate that EGF plays an important role in cancer development but not as an “initiating” agent (14) although a recent multicenter investigation demonstrated that the incidence of cancer was comparable between subjects treated with EGF and control subjects treated only with sulfadiazine. Furthermore, this incidence did not differ from the national incidence. All animal species subjected to long-term systemic administration of EGF show dose-dependent and reversible hyperplasia of epithelial organs without alterations in the phenotypic differentiation of cells. No pre-malignant histotypic markers were identified (13).

In conclusion, in this case, a topical therapy based on EGF was utilized on a premature neonate with benefit. This therapy was effective and rapid, resulting in complete healing of the pressure injury. Further clinical studies are necessary to confirm the efficacy of this new therapy in such a unique population as premature infants, where skin injuries are an extremely common issue.

## Data Availability

The original contributions presented in the study are included in the article/Supplementary Material, further inquiries can be directed to the corresponding author.
